# The complete mitochondrial genome of *Haemaphysalis montgomeryi*

**DOI:** 10.1080/23802359.2021.1945976

**Published:** 2021-07-14

**Authors:** Xinyan Lu, Dandan Jiang, Chunhong Du, Xing Yang

**Affiliations:** a Integrated Laboratory of Pathogenic Biology, College of Preclinical Medicine, Dali University, Dali, PR China; b Yunnan Institute of Epidemic Diseases Prevention and Control, Xiaguan District, Dali, Yunnan, PR China

**Keywords:** *Haemaphysalis montgomeryi*, mitogenome, phylogeny

## Abstract

The complete mitogenome of *Haemaphysalis montgomeryi* is reported for the first time in this study. The mitochondrial genome is 14,681 bp in length and includes 13 protein-coding genes, 2 ribosomal RNA genes, 22 transfer RNA genes, and 1 control region. The phylogenetic relations base on the maximum-likelihood (ML) method show that *H. montgomeryi* and the other members of the genus *Haemaphysalis* constitute a monophyletic group, confirming that *H. montgomeryi* belongs to the genus *Haemaphysalis.*

The tick *Haemaphysalis montgomeryi* (Nuttall, 1912) (Acari: Ixododae) has been recorded as a ectoparasite, which commonly parasitizes domestic animal, cattle, goat, sheep, buffalo and dog, and occasionally humans (Pun et al. 2018). *Haemaphysalis montgomeryi* is distributed in the subtropical and lower temperate levels of the Himalayas in West Pakistan, Nepal, India and China. In China, it is distributed in Sichuan, Guizhou, Tibet and Yunnan (Hoogstraal et al. 1966). Molecular features of complete mitogenomes have been reported in some ticks, but not for *H. montgomeryi*. We sequence the complete mitogenome of *H. montgomeryi* and present a classification of the genus *Haemaphysalis* (Chang et al. 2019).

Adult *H. montgomeryi* were collected (*n* = 2) from the body of a sheep, Jianchuan City, Yunnan Province, China (26°53′N, 99°88′E), in August 2018. The collected specimen was transported and stored at the Parasitological Museum, Dali University (Url: http://www.dali.edu.cn/jcyxy/xkpt/jcyxsyjxzx/6431.htm, Contact person: Xing Yang, yang08220013@163.com) under the voucher number: DLUP1808. The genomic DNA was isolated by the standard phenol–chloroform extraction procedure, and stored at −20 °C until use. Whole mitochondrial genome sequencing used the whole-genome shotgun method and was conducted on the Illumina NovaSeq platform by Shanghai Personal Biotechnology Co, Ltd, Shanghai, China. The mitogenome of *H. montgomeryi* was assembled using A5-miseq v20150522 software and SPAdesv3.9.0 software (Coil et al. 2015). Eventually, genome components annotation was retrieved using the MITOS web server.

The complete mitogenome of *H. montgomeryi* was 14,618 bp (GenBank accession no. MW751681), encoding 13 protein-coding genes (*atp*6, *atp*8, *cytb, nad*4L, *cox*1-3, and *nad*1-6), 2 ribosomal RNA genes, 22 transfer RNA genes, and 1 control region (Figure 1). The arrangement of the *H. montgomeryi* was identical with that of hard ticks (Thomas et al. 2013). The *H. montgomeryi* mitochondrial genome encoded 3620 amino acids in total. The overall base composition of the *H. montgomeryi* mitogenome was determined to be 39.04% T, 12.59% C, 39.42% A, and 8.95% G. The lengths of *H. montgomeryi* small subunit ribosomal RNA and large subunit ribosomal RNA were 709 bp and 1247 bp, respectively. The size of 22 tRNAs ranged from 53 bp (tRNA-Cys) to 68 bp (tRNA-Gln). The control region (253 bp) with 65.61% A + T content was placed between 12S rRNA and tRNA-Ile.

**Figure 1.**
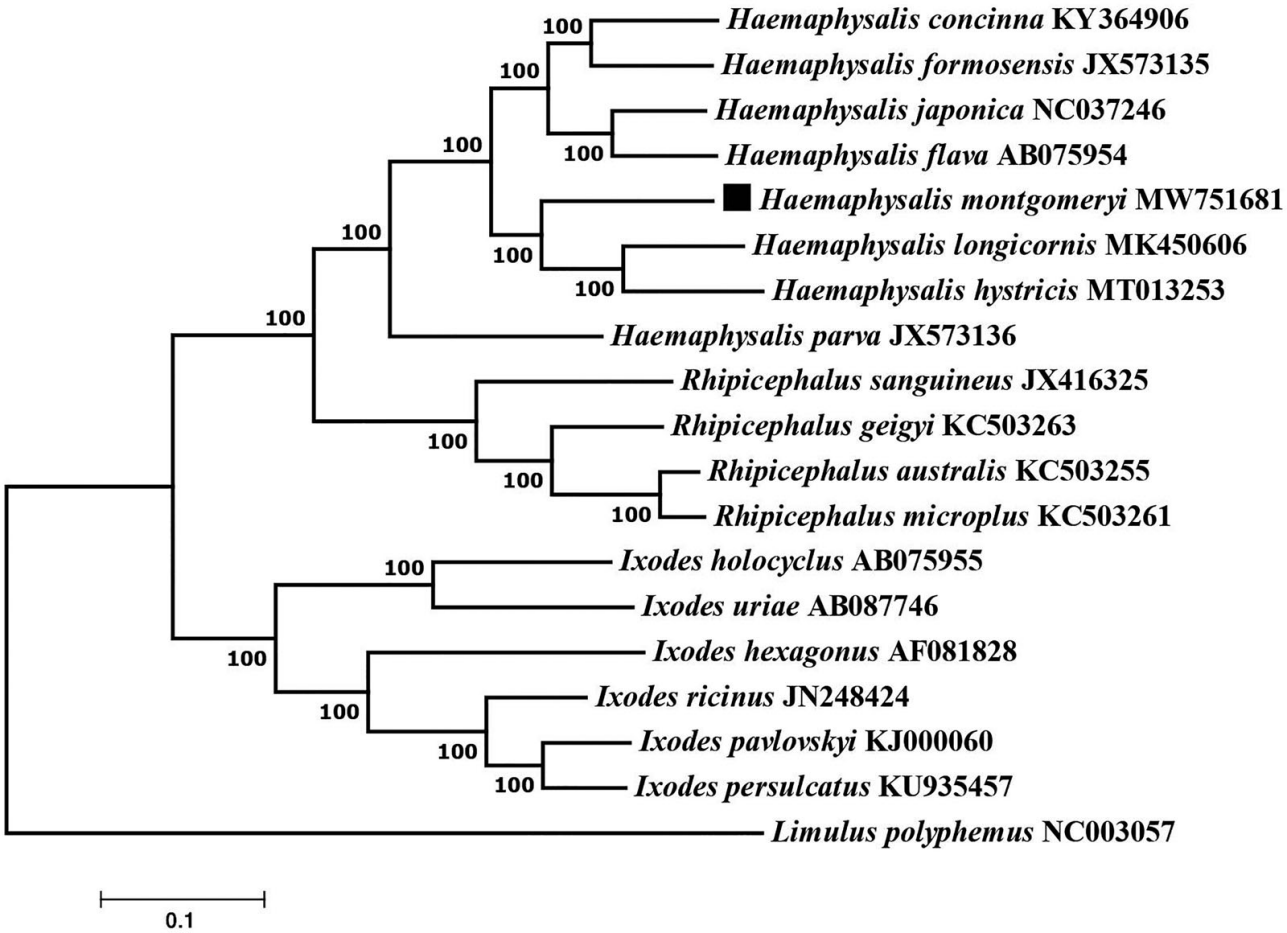
Phylogenetic relationships of *Haemaphysalis montgomeryi* and other species based on mitochondrial sequence data.

The concatenated amino acid sequences of 13 protein-coding genes were analyzed with the maximum-likelihood method based on the Tamura-Nei model with 1000 bootstrap replicates. The phylogenetic analysis included 18 published mitogenomes from *Ixododae* and *Limulus polyphemus* (NC003057) as an outgroup. Evolutionary analyses were conducted in MEGA7.0 software. The phylogenetic analysis shows that the *H. montgomeryi* and the others of genus *Haemaphysalis* are clustered into one clade with high statistical support, confirming that *H. montgomeryi* belong to the genus *Haemaphysalis.* The *H. montgomeryi* complete mitogenome provides a valuable resource for further studies on species identification of the tick genus *Haemaphysalis*.

## Data Availability

The data that support the findings of this study are openly available in GenBank of NCBI at https://www.ncbi.nlm.nih.gov/, reference number MW751681. The associated BioProject, SRA, and Bio-Sample numbers are PRJNA730208, SRR14601628, and SAMN19223702, respectively.
